# The Effects of Varying Degree of MWCNT Carboxylation on Bioactivity in Various In Vivo and In Vitro Exposure Models

**DOI:** 10.3390/ijms19020354

**Published:** 2018-01-25

**Authors:** Raymond F. Hamilton, Zheqiong Wu, Somenath Mitra, Andrij Holian

**Affiliations:** 1 Department of Biomedical and Pharmaceutical Sciences, Center for Environmental Health Sciences, University of Montana, Missoula, MT 59812, USA; ray.hamilton@mso.umt.edu; 2 Department of Chemistry and Environmental Science, New Jersey Institute of Technology, Newark, NJ 07102, USA; zw52@njit.edu (Z.W.); Somenath.Mitra@njit.edu (S.M.)

**Keywords:** MWCNT, macrophage, autophagy, carboxylation, dark-field imaging

## Abstract

Functionalization has been shown to alter toxicity of multi-walled carbon nanotube (MWCNT) in several studies. This study varied the degree of functionalization (viz., amount of MWCNT surface carboxylation) to define the relationship between the extent of carboxylation and effects in a variety of in vitro cell models and short-term ex vivo/in vivo particle exposures. Studies with vitamin D_3_ plus phorbol ester transformed THP-1 macrophages demonstrated that functionalization, regardless of amount, corresponded with profoundly decreased NLRP3 inflammasome activation. However, all MWCNT variants were slightly toxic in this model. Alternatively, studies with A549 epithelial cells showed some varied effects. For example, IL-33 and TNF-α release were related to varying amounts of functionalization. For in vivo particle exposures, autophagy of alveolar macrophages, measured using green fluorescent protein (GFP)- fused-LC3 transgenic mice, increased for all MWCNT tested three days after exposure, but, by Day 7, autophagy was clearly dependent on the amount of carboxylation. The instilled source MWCNT continued to produce cellular injury in alveolar macrophages over seven days. In contrast, the more functionalized MWCNT initially showed similar effects, but reduced over time. Dark-field imaging showed the more functionalized MWCNTs were distributed more uniformly throughout the lung and not isolated to macrophages. Taken together, the results indicated that in vitro and in vivo bioactivity of MWCNT decreased with increased carboxylation. Functionalization by carboxylation eliminated the bioactive potential of the MWCNT in the exposure models tested. The observation that maximally functionalized MWCNT distribute more freely throughout the lung with the absence of cellular damage, and extended deposition, may establish a practical use for these particles as a safer alternative for unmodified MWCNT.

## 1. Introduction

Multi-walled carbon nanotubes (MWCNTs) are a family of engineered nanomaterials consisting of concentric tubes of graphite with properties that are particularly conducive to mechanical and electrical applications. These materials are currently mass produced and are found in a variety of materials, ranging from composites, storage devices, pharmaceutical drug deliveries, optics, and engineering products [1]. Further, the use of surface modifications has allowed the expansion of relevant applications and is a useful mechanism for exploring and reducing the innate toxicity of MWCNTs [2,3]. Nonetheless, rapid advancement in the field of engineered nanomaterials is limited by concerns of potential toxicities (more broadly defined: bioactivity refers to toxicity and/or induction of acute/chronic inflammation) and long-term health consequences as these materials become more commonplace within our environment and casual internalization in humans is realized. As such, occupational and environmental exposures are expected to primarily affect the lung, as inhalation is the most logical route for MWCNT exposure [4].

The toxic properties of MWCNTs have been documented in numerous cell and animal exposure models, with particle length and rigidity mimicking asbestos fibers being the properties most commonly associated with harmful interactions [5]. However, in addition to length and rigidity, the presence of metal contaminants such as Fe and Ni [6], as well as properties involved in agglomeration likely contribute to MWCNT-associated pathogenesis [7] MWCNT-induced pulmonary fibrosis is a primary concern, with the best-described paradigm involving activation of the NLRP3 inflammasome following exposure, which is intimately involved in the activation of caspase-1 and release of interleukin (IL)-1β and IL-18 [8]. Further, gene expression analyses have shown impacts in pathways involved in carcinogenesis, such as cell death, cell proliferation, and free radical scavenging in mice exposed to MWCNTs [9]. While the exact mechanisms and relevant physicochemical properties remain to be fully defined, there is a clear need to further develop and test modified MWCNTs with better safety profiles.

Functionalization (i.e., chemical modification of the surface) of MWCNTs via carboxylation, which increases water solubility, has been shown to reduce toxicity in normal human liver cells [10,11] and liver indices of toxicity were systematically lower with increasing functionalization of MWCNTs [12]. In the lung, alveolar macrophages (AM) are responsible for clearing particle debris and are clearly involved in uptake and removal of MWCNTs of any variant [13]. However, it is not fully understood how lung epithelial cells interact with engineered nanomaterials in general and MWCNTs specifically. Most studies examining the effects of MWCNTs on epithelial cells have been conducted in cell lines. These studies have obvious drawbacks, as they isolate the cell type and particle, while ignoring any potential interaction occurring in the complex environment of the lung. Nonetheless, in human pulmonary epithelial cells, exposure to carboxylated MWCNTs resulted in microvilli modifications that were hypothesized to affect cellular functions, such as production and secretion of surfactant [14]. Further, in human alveolar A549 epithelial cells, carboxylated MWCNTs were confined within cytoplasmic vesicles, whereas pristine MWCNTs were found free in the cytoplasm as well as within vesicles [15]. Carboxylation has also been shown to increase MWCNT binding to bovine serum albumin (BSA) [16], suggesting the protein corona has an impact on the biocompatibility of functionalized MWCNTs.

Exposure to carboxylated MWCNTs clearly results in a distinct biological response that differs from that of unmodified MWCNTs. However, it remains unclear how the degree of carboxylation contributes to these observed differences. The hypothesis of this study is that increasing carboxylation on MWCNTs will decrease toxicity and the effects will be dependent on the degree of carboxylation. In addition, increasing carboxylation on MWCNTs will change the way the particles are taken up by the lung tissue indicative of surface alterations. In vitro and in vivo studies were conducted to evaluate the impacts on two key cell types that would be most likely involved, macrophages and epithelial cells. The results of this study will contribute to the body of knowledge currently used to improve the safety and biocompatibility of engineered nanomaterials, with a focus on MWCNTs.

## 2. Results 

### 2.1. Functionalized Particle Characterizations

**Figure 1:**
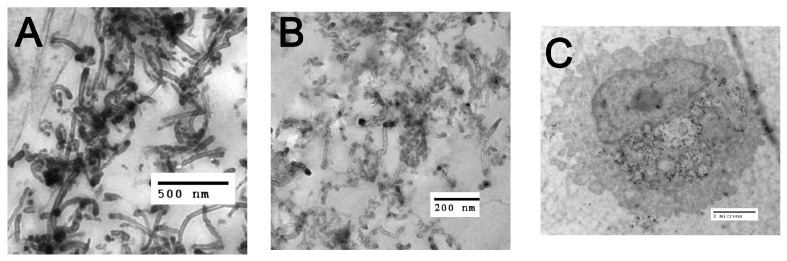
TEM images of particles and macrophage ingestion of MWCNTs: (**A**) TEM of source raw MWCNT; (**B**) TEM of carboxylated f-MWCNT_16.2_; and (**C**) TEM of macrophage internalizing source raw MWCNT (50 μg/mL) after 4 h.

The morphology of MWCNTs is shown in TEM image (Figure 1A). Contrasted with the carboxylated MWCNTs shown in Figure 1B, the source particle appears more aggregated and denser than the derivative f-MWCNTs, consistent with the information contained in Table 1. The elemental analysis of MWCNTs and f-MWCNTs were measured by EDX (Table 2). The oxygen content increased significantly with the treatment time from 5 to 40 min, but changed slightly for longer times. Residual Ni catalysts were present in the source MWCNTs, which decreased during treatment of the source MWCNTs to generate the f-MWCNTs from 5 and 10 min and after 20 min no Ni was detected. The ratio between carbon and oxygen (C:O) was calculated based on EDX data. The ratio decreased from 26.5 to 16.4 as the treatment time increased from 5 to 40 min. There was no further significant change beyond 40 min. The f-MWCNTs with different treatment times were referred to as f-MWCNT_38.4_, f-MWCNT_23.8_, f-MWCNT_20.6_, f-MWCNT_16.2_, f-MWCNT_15.7_, f-MWCNT_14.9_, and f-CNT_14.7_, based on the C:O ratio. The specific surface area (SSA) of samples (Table 2) increased from 174 to 266 m^2^/g with treatment time from the source MWCNTs to 40 min with little change beyond 40 min. Detailed methodology for how these f-MWCNT were developed can be found in Wu et al. [17].

### 2.2. In Vitro Effects of Varied Carboxylation

**Figure 2:**
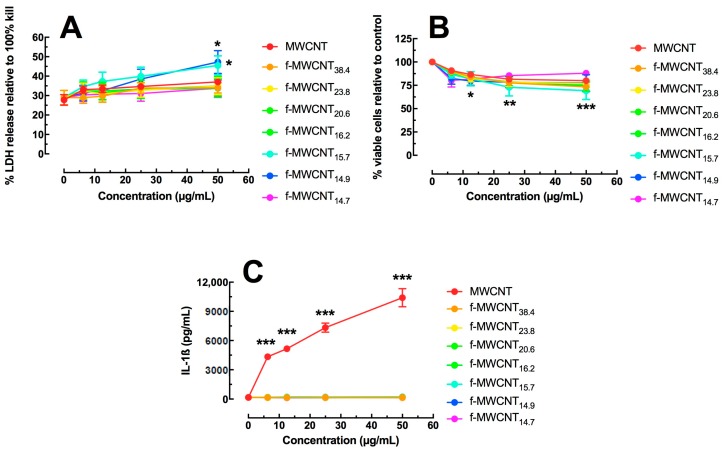
Effect of functionalized MWCNT on THP-1 macrophage-like cells in an in vitro 24 h culture. (**A**) Cell toxicity determined by LDH activity in cell media. Data expressed as mean LDH percent ± SEM compared to 100% cell death. (**B**) Cell toxicity determined by MTS assay. Data expressed as mean ± SEM viability relative to control cultures (0 μg/mL). (**C**) IL-1β release expressed as mean ± SEM pg/mL. Asterisks *** indicate *p* < 0.001, ** *p* < 0.01, or * *p* < 0.05 compared to 0 μg/mL concentration.

The transformed human monocytic cell line THP-1 was used to assess differential f-MWCNT toxicity and bioactivity after a 24 h exposure using an in vitro cell culture model. Once transformed by vitamin D_3_ exposure, the THP-1 cell is phenotypically similar to a mature macrophage cell. Two methods were used for determination of cytotoxicity: LDH release assay and the MTS assay. Figure 2A shows LDH assay results for dose response evaluations of the original and various functionalized MWCNT. At the highest particle concentrations, only two f-MWCNTs (f-MWCNT_15.7_ and f-MWCNT_14.9_) showed significant toxicity. These f-MWCNTs were two of the more functionalized MWCNT. In contrast, all MWCNTs showed a dose-dependent cytotoxicity compared to no particle control by MTS assay starting at the 12.5 μg/mL concentration (Figure 2B). The one exception was the most functionalized CNT (f-MWCNT_14.7_), which showed no toxicity within this dose range. Regardless of the assay, there was no more than 2% to 20% cell death at the highest MWCNT concentration, indicating that the toxicity was moderate, even at the highest dose tested. The MTS assay may be more reliable when using functionalized carbon because the particles get caught in suspension and can affect the optical density results in the LDH assay [18]. This is not a problem with the MTS assay because the supernatant is completely removed and replaced with fresh media containing the MTS reagent.

Functionalized-MWCNT bioactivity was determined by IL-1β release in the transformed THP-1 cells as a proxy measure of NLRP3 inflammasome activation. LPS activates the NF-κB pathway to form pro-IL-1β (see Figure 1C). Significant THP-1 IL-1β release in vitro in response to particle exposure has been correlated with acute in vivo inflammatory potential (neutrophil recruitment) for inhaled engineered nanomaterials [18,19,20]. Figure 2C illustrates IL-1β release at 24 h with MWCNT exposure. Only the source MWCNT (non-functionalized CNT) exposure produced significant dose-dependent increases in IL-1β. This result confirmed other work showing that f-MWCNT did not inflammatory stimulate the NLRP3 inflammasome in vitro [2,3]. The new information in Figure 2C indicated the degree of functionalization was not a factor, and even the least functionalized MWCNT was sufficient to create an inert MWCNT with regard to NLRP3 inflammasome activation. Stated another way, the amount of carboxylation on the MWCNT had no effect with regard to NLRP3 inflammasome activity, as the least functionalized MWCNT was identical to the most functionalized MWCNT.

**Figure 3:**
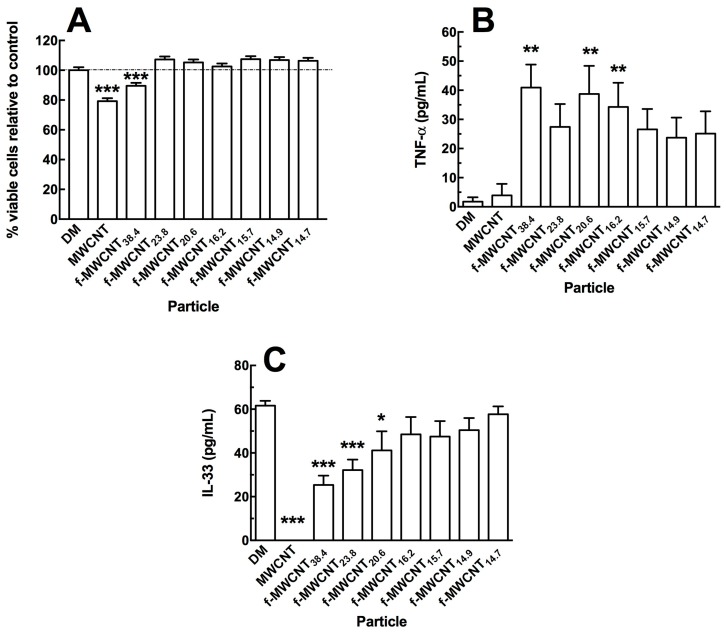
Effect of functionalized MWCNTs on A549 lung epithelial cells in an in vitro 24 h culture. (**A**) Cell toxicity determined by MTS assay. All particle concentrations were at 50 μg/mL. Data expressed as mean ± SEM optical density at 490 nm. (**B**) TNF-α release expressed as mean ± SEM pg/mL. (**C**) IL-33 release expressed as mean ± SEM pg/mL. Asterisks *** indicate *p* < 0.001, ** *p* < 0.01, or * *p* < 0.05 compared to appropriate control condition (no particle cultures (DM) or source MWCNTs (B only)).

During inhalation, in addition to macrophages, lung epithelial cells would be the most likely cell type to come in contact with inhaled MWCNTs. Therefore, the human A549 cell line was used to determine potential cytotoxicity and bioactivity in response to the differentially functionalized MWCNTs using the same 24 h in vitro culture exposure protocol. The MTS assay was used to determine cytotoxicity and two cytokines (IL-33, and TNF-α) were used to determine potential bioactivity. The A549 cells were adherent prior to particle exposure to better mimic the exposure conditions in the lung airway lining. Figure 3A shows the relative cytotoxicity of the source MWCNTs and the various f-MWCNTs, each at 50 μg/mL. Only the source MWCNTs and the least functionalized MWCNTs showed significant toxicity compared to no-particle control cultures. All other f-MWCNTs showed no significant differences to the no-particle control cultures. The cell death caused by the source MWCNTs was approximately 25%. These results are similar to earlier reports that functionalization reduced toxicity of MWCNTs in vitro [2,3]. 

Two potent cytokines were monitored after 24 h in the A549 culture supernatants. Figure 3B shows TNF-α release following MWCNT exposures. All of the f-MWCNT exposed A549 cells showed increased TNF-α release. In contrast, the baseline IL-33 release (Figure 3C) was eliminated by the source MWCNT, but returned to normal control culture amounts depending on the degree of MWCNT functionalization. This was the only cytokine that showed sensitivity to the amount of carboxylation on the f-MWCNT. Release of IL-1α, IL-6 and HMGB_1_ were also examined, however no effect of carboxylation on MWCNTs was apparent.

### 2.3. In Vivo Effects

**Figure 4:**
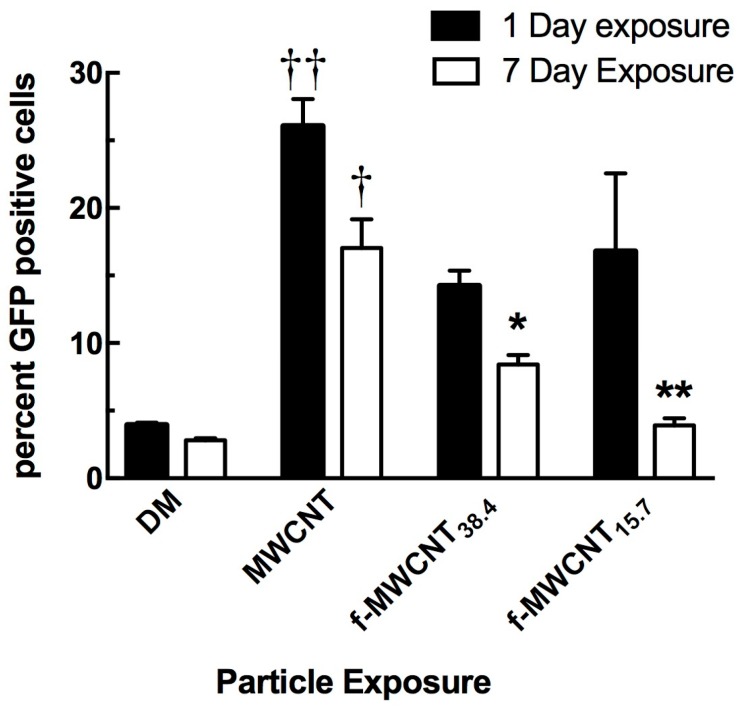
MWCNT functionalization effects autophagy as seen by GFP LC3 detection following particle instillation. One-day particle instillation of 50 μg indicated by dark bars. The seven-day instillation of 50 μg indicated by white bars. Data are expressed as mean ± SEM percent macrophages expressing LC3 green fluorescent protein, which indicates the presence of autophagic activity. Functionalization of 38.4 is 5 min of processing and 15.7 is 60 min of processing. Daggers †† indicate significance at *p* < 0.01, or † indicating significance at *p* < 0.05 compared to corresponding DM vehicle negative controls. Asterisks ** indicate significance at *p* < 0.01, or * indicating significance at *p* < 0.05 compared to corresponding MWCNT source controls. *n* = 3–4 mice per group.

Since nanomaterials have been shown to induce autophagy [19,20,21], an autophagy assay was chosen as a method to determine particle-induced in vivo bioactivity in the lung. These studies used C57Bl/6 mice with GFP-tagged LC3 to observe activation of the autophagy pathway with the source MWCNTs relative to a couple of f-MWCNT variants. As described in Methods and Materials, analysis of autophagy was conducted on lavaged cells by flow cytometry. The isolated cells were alveolar macrophages as predetermined by flow cytometry gating parameters. One-day post exposure was selected because all particles create some acute inflammatory response with lung exposure. The seven-day time point was selected because it is typically the maximal acute inflammatory response time for MWCNT lung exposures [2,6]. As expected, all MWCNT variants activated autophagy 24 h post-exposure (Figure 4). Only the source MWCNT was statistically significant compared to the DM baseline control. However, by Day 7, autophagy was starting to recede and a clear pattern was beginning to emerge. Activation of autophagy remained highest for the source MWCNTs, and then was significantly less for the f-MWCNT_38.4_ and completely resolved to baseline levels for the f-MWCNT_15.7_ exposure. This was indicative of an autophagic response to MWCNTs dependent on the degree of carboxylation with the most carboxylated MWCNTs being the least activating particle in the mouse lung over time (Figure 4).

**Figure 5:**
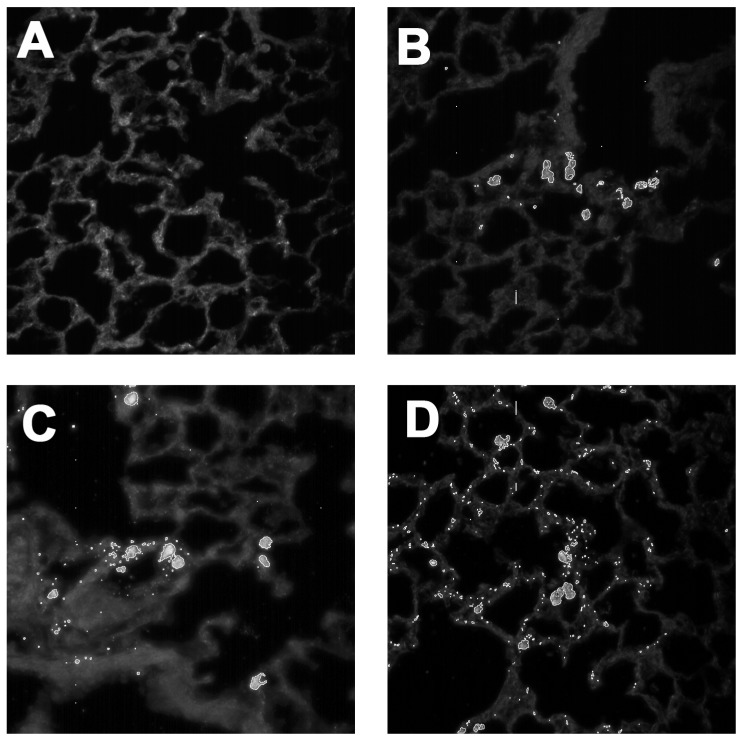
CytoViva dark-field images (400×) using Particle Fitting Function to locate MWCNT in lung tissue following a three-day particle exposure: (**A**) no particle dispersion media control; (**B**) source MWCNTs isolated mainly in alveolar macrophages. (**C**) carboxylated MWCNT_20.6_ (20 min) starting to show some distribution in the lung lining in addition to AM cell collection; and (**D**) maximally carboxylated MWCNT_14.7_ (120 min) demonstrates particle dispersion throughout the lung lining indicating epithelial cell involvement.

The above studies demonstrated that MWCNTs were effective in activating the autophagy pathway, with a decreased response as functionalization increased. Therefore, it was important to determine whether the in vivo distribution of the various MWCNTs in the lung could account for the observed response. Examination of particle-exposed lung tissue was conducted using Balb/c mice that were exposed to the MWCNT variants for three days. It was anticipated that interaction of MWCNTs would occur primarily with lung macrophages. However, one unexpected observation was that, as the amount of carboxyl groups increased on the f-MWCNTs, small portions of the f-MWCNTs were present on cells other than macrophages. Although difficult to see in bright field (even at high magnification), smaller derivative particles were found in the lung lining tissue epithelial surface. Figure 5A shows normal lung tissue at high magnification. Figure 5B, in contrast, shows the normal response to the source MWCNTs, as the particles were primarily taken up by AM. Unlike these two morphologies, Figure 5C shows deposition on epithelial cells of the f-MWCNT_20.6_. 

This was first noticed with the f-MWCNTs treated for 60 min, but it became more obvious with the more functionalized MWCNTs (treated for 90 or 120 min). Using CytoViva modified dark field microscopy, with the particle deposition highlighted, the extent of the f-MWCNT deposition was obvious (Figure 5D of the f-MWCNT_14.7_). This indicated that a significant portion of the f-MWCNTs had some deposition of the particle in the lung lining epithelial surface. It was clear, however, that the degree of functionalization was critical to the amount of particle that deposits on the epithelial cells of the lung. Greater degree of carboxyl groups on the MWCNTs was indicative of more epithelial particle deposition following instilled lung exposures. 

## 3. Discussion

It is widely established that functionalization, particularly carboxylation, can reduce the harmful effects of MWCNT exposure in various models [2,3,12,22,23]. What has not been determined is how much carboxylation is necessary to affect the bioactivity of MWCNTs, and whether there are differential effects on key lung cells with degree of carboxylation. Several physical changes in MWCNTs occur when the particles are carboxylated. The f-MWCNTs are more hydrophilic [16], less aggregated [22,24], create more unstable protein corona [25], and are more soluble [16] than the source MWCNTs. In addition, size, surface charge and zeta potential are altered, resulting in unique protein binding and protein corona upon carboxylation [24,25]. The practical reason for the placement of carboxyl groups on the surface of MWCNTs is to increase the covalent binding efficiency of the MWCNT surface. This allows for attachment of various materials (e.g., drugs, metals, amines, etc.) for potential therapeutics and other applications [1,26,27]. When discussing the effects of varying degrees of carboxylation, it is important to distinguish the general effects of carboxylation from the specific effects of varying degrees of carboxylation on the particle.

The main general biological effects of carboxylation are to reduce particle toxicity, bioactivity, inflammation, cell damage and pathological potential. Several studies have described these effects [2,3,10,11,12,28,29]. Similar results were observed in this study with the THP-1 (human macrophage model) and A459 (human lung epithelial cell model) cell lines response to the f-MWCNT variants. The MTS assay in the A549 cell showed that f-MWCNTs, regardless of degree of carboxylation, significantly reduced the toxicity compared to the source MWCNTs (Figure 3A). In the THP-1 cell line, all f-MWCNT variants were all slightly toxic with no significant difference compared to the source MWCNTs (Figure 2A,B), but the big differential was seen in the detection of NLRP3 inflammasome activation. In this case, the original MWCNTs produced a significant concentration-dependent increase in IL-1β release, which was totally absent with all f-MWCNT variant exposures in the THP-1 cell (Figure 2C). In addition, the increased release of TNF-α in the A549 cells could be due to a general effect of carboxylation, or alternatively could be due to the increased presence of endotoxin as a result of the preparation procedures (Figure 3B). However, endotoxin may not be responsible since there was no difference in IL-6 release and no release of IL-1β by macrophages, which are both stimulated by endotoxin.

There were some unique outcomes as a result of differential carboxylation. For example, the release of IL-33 in the particle-exposed A549 cells showed a distinct effect of increased carboxylation on the f-MWCNTs, as the lower carboxylation produced results similar to the source MWCNTs, which increased proportionally to the amount of carboxylation in a dose-dependent manner (Figure 3C). Similarly, the in vivo exposures and detection of autophagy showed that the alveolar macrophages had high rates of activation of autophagy by all particles tested, but the most functionalized MWCNT-exposed AM were faster to recover, with the source MWCNTs still causing autophagy at seven days post-exposure (Figure 4). Autophagy is an important biological response that has been shown to be increased by a variety of nanomaterials [21,30]. This study demonstrated that source MWCNTs were the most potent, but, as carboxylation increased, the impact on autophagy decreased. A potential explanation for this effect may be due to differential interactions of the source MWCNTs compared to the differentially-carboxylated MWCNTs with the inner membrane of phagolysosomes once the particles have been phagocytosed. Consequently, this suggests that a more hydrophilic surface would cause less membrane damage to phagolysosomes and less lysosome membrane permeability [19,25,31].

An important distinction regarding the degree of carboxylation on f-MWCNTs was established in the distribution of instilled materials (Figure 5A–D). It was clearly evident in modified dark field imaging that maximally carboxylated f-MWCNTs distributed differently throughout the murine lung tissue after a three-day particle exposure compared to the source MWCNTs and minimally f-MWCNTs. This is important for several reasons. First, the principle interaction of MWCNTs is with the AM, responsible for uptake and clearance. This typically produces a number of inflammatory signals that can lead to a sustained response and even disease depending on the frequency and amount of exposures [32]. This could potentially explain why f-MWCNTs are less inflammatory, autophagy inducing and pathogenic. Second, the potential interaction with epithelial cells creates a therapeutic target in the lung, as f-MWCNTs can be modified to deliver drugs to areas of inflammation independent of AM interference [26,33]. Lastly, the differential uptake of the f-MWCNTs suggests a surface recognition mechanism most likely due to different proteins/lipids coating the different f-MWCNTs once exposed to lung surfactant or culture media in the case of in vitro experiments [14,16,22,25]. 

There are several proposed reasons for why f-MWCNTs are taken up by epithelial cells. The first reason is the relative decrease in aggregation and the smaller size that results from carboxylation [14,34]. This study supports that conclusion with one caveat. Our data imply that there is actually an interaction between size and degree of carboxylation, with the more functionalized particle being taken up the most by epithelial cell in vivo. The reduction in size was relatively constant across carboxylation degrees, as shown in Table 1. That alone cannot account for the increased uptake of particle. In addition, studies have demonstrated that different protein coronas develop around carboxylated MWCNTs in contrast to source MWCNTs [16,22]. Typically, more hydrophilic proteins would stick to the f-MWCNTs compared to source MWCNTs [35,36]. Unfortunately, this process has not been systematically studied with regard to varying the amount of carboxylation, so it is difficult to know if this was a factor in this study. Lastly, surface charge and zeta potentials can be altered as a result of functionalization. Several studies indicate that this can make the particle variants more or less interactive with different cell types [15,16,34]. However, this may not appear to have been the case in this study, as there was no change in zeta potential with any MWCNT variant tested. The size/functionalization degree hypothesis works best for the results described in this study, although other factors cannot be discounted, as they could not be systematically eliminated as a possibility in this work.

With regard to biological testing of the various f-MWCNTs, changing the degree of carboxylation had very few effects in both THP-1 and A549 cell line exposure models. The biggest differences in cytotoxicity and NLRP3 inflammasome activation were always between the f-MWCNTs, in general, and the source or non-functionalized MWCNTs. This was consistent with previous findings [2,3], which demonstrated that functionalization of MWCNTs created a relatively inert nanoparticle in several particle exposure models with regard to toxicity and inflammatory potential. This study showed that the attenuated toxicity and bioactivity produced by f-MWCNTs was not dependent on the amount of functionalization. However, the degree of MWCNT functionalization had a significant effect on in vivo metrics, including the induction and resolution of autophagy contrasted with the deposition of particles in the lung, which demonstrated an alternative uptake mechanism with the more functionalized MWCNTs.

## 4. Materials and Methods

### 4.1. Preparation of the Functionalized-MWCNTs

Multiwall carbon nanotubes (MWCNTs) (OD 20–30 nm, length 10–30 μm, purity > 95%) were purchased from Cheap Tubes Inc. (Cambridgeport, VT, USA), and all other chemicals were purchased from Sigma Aldrich (St. Louis, MO, USA) with purity higher than 95%. The synthesis of the functionalized MWCNTs (referred to as f-MWCNTs) was carried out in a Microwave Accelerated Reaction System. Pre-weighed amounts of MWCNTs combined with a mixture of concentrated H_2_SO_4_ and HNO_3_ were added to reaction chambers. Carboxylation was initiated by microwave radiation at 140 °C from 5 to 120 min. Subsequently, the samples were cooled to room temperature, vacuum filtered with 10 μm filters and washed with Milli-Q water until the wash was neutral pH. The f-MWCNTs were then dried in a vacuum oven at 70 °C until constant weight of the sample was achieved.

### 4.2. Characterization of the Functionalized-CNTs

#### 4.2.1. Transmission Electron Microscopy of Particles and AM 

Samples were fixed in 2.5% EM grade glutaraldehyde in cacodylate buffer at pH 7.2. The samples were then rinsed in dH_2_O and resuspended in 1% osmium tetroxide for 1 h and rinsed in dH_2_O. The cell pellets were embedded in epoxy after drying using a series of increasing ethanol concentrations. Thin sections were cut then stained for 30 min at room temperature with 2% uranyl acetate. The sections were then rinsed in dH_2_O and stained with Reynolds lead citrate for 5 min. A Hitachi H—7100 transmission electron microscope set at 75 kV was used to image the samples. For isolated AM TEM, the AM were exposed to 50 μg/mL MWCNT variants for 4 h in a suspension culture. They were then centrifuged in a microfuge, washed once in PBS and processed as described above. 

#### 4.2.2. Particle Size and Zeta Potentials

Hydrodynamic size and surface average zeta potentials were measured in culture media at 25 °C using a Malvern Zetasizer nano Zen 3600 (Malvern Instruments, Worcestershire, UK) at a 90° detector angle. Endotoxin contamination was determined by washing/sonicating 1 mg/mL MWCNTs in endotoxin-free water for 30 min followed by centrifugation at 16,000× g for 15 min prior to assay. The assay (ToxinSensor) was performed on the isolated supernatant according to the manufacturer’s protocol (GenScript, Piscataway, NJ, USA). Aggregate hydrodynamic size was expressed as nm ± the standard deviation. The zeta potential was expressed as mV ± the standard deviation. The endotoxin contamination was expressed as ng endotoxin/5 μg of MWCNT, which represents the largest mass of MWCNTs added to the cells (50 μg/mL). Results can be found in Table 1.

#### 4.2.3. Surface Area Assessment

Scanning electron microscope (SEM) elemental analysis was employed for surface area assessment (SSA). The MWCNT sample variants were processed on a LEO 1530 VP scanning electron microscope equipped with an energy-dispersive X-ray analyzer. The specific SSA of the particles were measured using a Quantachrome NOVA 3000 series (Model N32-11) High Speed Gas Sorption Analyzer at 77.40 K. The particles were heated and degassed at 300 °C in a vacuum oven for three hours prior to the SSA measurements. Results can be found in Table 2.

#### 4.2.4. CNT Suspensions

All nanotubes were weighed and suspended in dispersion media (DM), which consisted of mouse serum albumin (Sigma, St. Louis, MO, USA; 1 mg/mL) and 1,2-dipalmitoyl-*sn*-glycero-3-phosphocholine (DSPC, Sigma, 1 μg/mL) in phosphate-buffered saline (PBS). Nanotube suspensions were sonicated for 5 min at 1/3 max power in a Qsonica cup-horn sonicator (Q500, Newtown, CT, USA) attached to a VWR circulating water-bath at 500 watts and 20 Hz (8000 Joules) at a stock concentration of 1 mg/mL (in vivo) or 5 mg/mL (in vitro).

### 4.3. Human THP-1 Cell Line Culturing

THP-1 cells, a human monocytic cell line obtained from ATCC, were suspended in RPMI media (MediaTech, Manassas, VA, USA) supplemented with 10% fetal bovine serum, 50 μM beta-mercapto ethanol, 1 mM sodium pyruvate, 250 ng/mL amphotericin B, and 100 U/mL penicillin and streptomycin (all supplements Media Tech, Manassas, VA, USA) in 75 cm^2^ flasks at 37 °C. The cells in suspension were differentiated into a macrophage-like cell by adding 150 nM Vitamin D_3_ (1α, 25-dihydroxy, EMD Millipore, Darmstadt, Germany) for 24 h. The semi-adherent cells were scrapped with a rubber policeman in the existing media (Corning, Corning, NY, USA). The cells were centrifuged at 400× g for 5 min, the resulting cell pellet was re-suspended in 1 mL of complete media, and a 40 μL sample was counted on a Z2 Coulter Counter (Beckman Coulter, Miami, FL, USA). The remaining cells were suspended at 1 × 10^6^ cells/mL, and a small amount of phorbol 12-myristate 13-acetate (5 nM PMA, Sigma) and lipopolysacharride (10 ng/mL LPS, Sigma) was added. Note: PMA co-stimulation serves to help transform THP-1 cells to be macrophage-like and to stimulate phagocytosis of the CNT. LPS was used to induce NF-κB translocation leading to pro-IL-1β synthesis for the NLRP3 inflammasome to cleave for IL-1β release in the transformed THP-1 model [18]. Cells, at a volume of 350 μL, were transferred in to 1.5 mL microfuge tubes. The MWCNT conditions were added from 5 mg/mL concentrated stock suspensions to the cells at a final concentration of 25 μg/mL. The MWCNT variants used a range of concentrations (0, 6.25, 12.5, 25, and 50 μg/mL). The resulting cell/particle suspension was mixed by pipette action. The mixed cell/particle suspensions were transferred to 96-well tissue culture plates at 100 μL per well in three pseudo-replicate cultures (100 × 10^3^ cells/well), and maintained for an additional 24 h. All cultures were kept in 37 °C water-jacketed CO_2_ (5%) incubators (ThermoForma, Houston, TX, USA). Viability and IL-1β levels were determined as described below. Three to four experimental replicates were done for each experiment.

### 4.4. Human A549 Cell Line Culturing

A549 cells, a human lung epithelial cell line obtained from ATCC, were suspended in modified F-12 media (MediaTech, Manassas, VA, USA) supplemented with 10% fetal bovine serum, 250 ng/mL amphotericin B, and 100 U/mL penicillin and streptomycin (all supplements Media Tech, Manassas, VA, USA) in 75 cm^2^ flasks at 37 °C. This cell line is adherent, so trypsin/EDTA was used to dislodge the cells for passing and/or experimental particle exposures. Suspended cells were centrifuged at 300× *g* for 4 min prior to counting, as described above. The A549 cells were seeded in 96-well plates at 15 × 10^3^ cells/well for 24 h prior to MWCNT exposure. At 24 h, the supernatants were removed and replaced with new media containing 50 μg/mL of a specific CNT in triplicate wells. The culture proceeded for an additional 24 h before viability (MTS only) and cytokine levels were determined as described below. Three to four experimental replicates were done for each experiment.

### 4.5. Toxicity Assays

Cell viability was determined by MTS assay using the CellTiter^96^ assay (Promega, Madison, WI, USA) according to the manufacturer’s protocol with a simple modification as described below. This assay used a colorimetric dye read by a colorimetric plate reader (Molecular Devices, Sunnyvale, CA, USA). To avoid optical density artifacts created by the presence of CNTs in the optical density values, steps were taken to remove the MTS reagent (transferring it into another plate) from the cell/particle mixture adhered to the plate bottom. The formation of bubbles was avoided and the plate was read at 490 nm. Data were expressed as percent viable cells relative to control cultures without particle (0 μg/mL). In addition, lactate dehydrogenase (LDH) was determined in 24 h culture supernatants by using CellTox^96^ assay (Promega, Madison, WI, USA) according to the manufacturer’s protocol. Data were expressed as percent LDH relative to 100% cell death (obtained from cultured detergent-lysed cells 30 min before assay).

### 4.6. Cytokine Assays

Human IL-1α, IL-1β, IL-6, IL-33 and TNF-α DuoSets were obtained from R&D Systems (Minneapolis, MN, USA) and ELISA assays performed according to the manufacturer’s protocol. Plates were read at 450 nm and data expressed as pg/mL.

### 4.7. Animals 

Balb/c and GFP-LC3 mice on C57Bl/6 background (2-months old, male) were housed in controlled environmental conditions (22 ± 2 °C; 30–40% humidity, 12-h light: 12-h dark cycle) and provided food and water ad libitum. All procedures were performed under protocols approved by the IACUC of the University of Montana (05017 AUP ID code, 21 July 2017). 

### 4.8. Lung Cell Isolation

Mice were euthanized by sodium pentobarbital (Euthasol™ Schering-Plough, Lot# 1JRR11V), and the lungs with the heart were removed. Lung lavage was performed using ice-cold PBS (pH 7.4). Lung lavage cells were isolated by centrifugation (400× *g*, 5 min, 4 °C) and cell counts obtained using a Coulter Z2 particle counter (Beckman Coulter, Miami, FL, USA).

### 4.9. Autophagy Determination In Vivo

Autophagy was examined since MWCNTs have been shown to induce autophagy and autophagy is a primary mechanism for removal of assembled inflammasomes [19,20,37] and sequestration of damaged organelles (e.g., permeabilized phagolysomes) [38,39]. GFP-LC3 mice were used for assessment of autophagy in vivo using methods described by Mizushima et al. [40]. GFP-LC3 mice were instilled as described above with (3) MWCNT variants: the source MWCNTs, the minimally carboxylated f-MWCNT_38.4_ (5 min) and a moderately carboxylated f-MWCNT_15.7_ (60 min). The no particle control was dispersion media alone. The exposed lungs were removed and lavaged to acquire the alveolar lung cells, one day and seven days post-particle exposure. These cells were suspended in PBS and run through a Flow Cytometer to determine the percent of GFP positive cells [21,40]. Analysis of fluorescent GFP/LC3 was accomplished using a FACSAria (BD Biosciences, San Jose, CA, USA) system. A 488-nm laser was used to excite the GFP tag and a 515/30 bandpass filter/PMT was used for GFP detection. Dead cells were excluded from analysis using DAPI co-staining excited by a 405-nm laser with detection in a 450/40 nm bandpass filter/PMT detector set. Data analysis was performed using FACSDiva software (BD Biosciences). AM were isolated 1 and 7 days following MWCNT exposure and incubated in PBS. Naïve AM were also isolated from DM-exposed mice following the same time course to establish a baseline control. 

### 4.10. Dark-Field Imaging

MWCNT deposition in lung tissue was documented by using a hyperspectral dark-field imaging system made by CytoViva (Auburn, AL, USA). Carbon nanotubes are difficult to isolate with conventional Spectral Angle Mapping (SAM) techniques, as the spectral libraries generated from the MWCNT do not differentiate well from the background tissue. An alternative process called Particle Fitting was used to make carbon particles more easily detectible in this system. This process used the relative intensity (brightness) of the particle in a dark-field image to determine what was particle and what was background. In addition, the particle or signal is encircled with a white line to amplify the presence of the signal. All images were captured at 400× and processing was done with ENVI software (v.4.8, CytoViva, Alburn, AL, USA). 

### 4.11. Statistical Analyses

Statistical analyses involved comparison of means using a one- or two-way *ANOVA* followed by Dunnett’s test or Sidak’s adjustment to compensate for increased type I error resulting from pair-wise mean comparisons. All probabilities were two-tailed unless otherwise stated. Statistical power was greater than 0.8. Statistical significance was defined as a probability of type I error occurring at less than 5% (*p* < 0.05). The minimum number of experimental replications was 3. Graphics and analyses were performed on PRISM (v.7, GraphPad, San Diego, CA, USA).

## Figures and Tables

**Table 1:** Agglomerate size (mean ± SD), zeta potential (mean ± SD), and endotoxin contamination of MWCNT in RPMI culture media after dispersion medium preparation.

Particle	Hydrodynamic Size (nm)	Zeta Potential (mV)	Endotoxin (ng/5 μg MWCNT)
MWCNT-_source_	396 ± 22.0	−10.3 ± 0.39	0.02
f-MWCNT_38.4_	169.1 ± 50.9	−12.7 ± 0.34	2.4
f-MWCNT_23.8_	57.6 ± 2.3	−10.4 ± 0.96	2.95
f-MWCNT_20.6_	67.0 ± 9.4	−11.1 ± 0.42	2.9
f-MWCNT_16.2_	44.2 ± 32.5	−11.5 ± 1.01	2.7
f-MWCNT_15.7_	272.8 ± 1.1	−11.1 ± 1.3	3.25
f-MWCNT_14.9_	234.6 ± 7.2	−10.2 ± 1.0	3.3
f-MWCNT_14.7_	41.8 ± 1.2	−11.5 ± 1.2	3.2

**Table 2:** Elemental analysis, C:O ratio, SSA of MWCNT and f-MWCNT.

Particle	Treatment Time (min)	% by Weight			C:O	SSA (m^2^/g)
		C	O	Ni		
MWCNT_source_	0	93.9	4.90	1.20	N/A	174
f-MWCNT_38_	5	90.1	9.06	0.84	13.3	220
f-MWCNT_24_	10	88.9	10.7	0.40	11.1	247
f-MWCNT_21_	20	88.2	11.8	-	10.0	246
f-MWCNT_16_	40	86.0	14.0	-	8.2	266
f-MWCNT_15_	60	85.2	14.8	-	7.7	263
f-MWCNT_15_	90	84.9	15.1	-	7.5	251
f-MWCNT_14_	120	84.4	15.6	-	7.2	271
